# Meeting report: Ocean ‘omics science, technology and cyberinfrastructure: current challenges and future requirements (August 20-23, 2013)

**DOI:** 10.4056/sigs.5749944

**Published:** 2014-03-15

**Authors:** Jack A Gilbert, Gregory J. Dick, Bethany Jenkins, John Heidelberg, Eric Allen, Katherine R. M. Mackey, Edward F. DeLong

**Affiliations:** 1 Argonne National Laboratory, Argonne, IL, USA & Department of Ecology and Evolution, University of Chicago, Chicago, IL; 2 Department of Earth and Environmental Sciences and Department of Ecology and Evolutionary Biology, University of Michigan, Ann Arbor, MI, USA; 3 Depts of Cell and Molecular Biology and Oceanography, University of Rhode Island, RI 02881; 4 University of Southern California, College of Letters, Arts and Sciences, Los Angeles, CA; 5 Scripps Institution of Oceanography and Division of Biological Sciences, University of California, San Diego, CA; 6 Woods Hole Oceanographic Institution, Marine Chemistry and Geochemistry, Woods Hole, MA; 7 Center for Microbial Oceanography, Research and Education (C-MORE), University of Hawaii, Manoa, Honolulu, HI

## Abstract

The National Science Foundation’s EarthCube End User Workshop was held at USC Wrigley Marine Science Center on Catalina Island, California in August 2013. The workshop was designed to explore and characterize the needs and tools available to the community that is focusing on microbial and physical oceanography research with a particular emphasis on ‘omic research. The assembled researchers outlined the existing concerns regarding the vast data resources that are being generated, and how we will deal with these resources as their volume and diversity increases. Particular attention was focused on the tools for handling and analyzing the existing data, on the need for the construction and curation of diverse federated databases, as well as development of shared, interoperable, “big-data capable” analytical tools. The key outputs from this workshop include (i) critical scientific challenges and cyber infrastructure constraints, (ii) the current and future ocean ‘omics science grand challenges and questions, and (iii) data management, analytical and associated and cyber-infrastructure capabilities required to meet critical current and future scientific challenges. The main thrust of the meeting and the outcome of this report is a definition of the ‘omics tools, technologies and infrastructures that facilitate continued advance in ocean science biology, marine biogeochemistry, and biological oceanography.

## Introduction

A large group of ocean scientists and oceanographers are now employing “’omics” approaches to characterize and quantify the nature, distribution and function of organisms in ocean ecosystems [1-3]. “’Omics” is defined here as the collective molecular or biochemical characterization of pools of biological molecules, such as genes and genomes, transcripts and transcriptomes, proteins and proteomes, and small molecules, metabolites and metabolomes, that together encode the structure and function of an organism or organisms, and can be used to explore their dynamics and flexibilities. The tools and datasets that encompass 'omics science are diverse, complex, and rapidly expanding, and require the construction, curation, and query of diverse federated databases, as well as the development of shared, interoperable, “big-data capable” analytical tools. Given the trajectory of “next generation” sequencing technologies, economics, and applications, this arena represents a major “big data challenge” for the ocean science community at large.

To discuss the ‘omic data challenges for ocean scientists, an NSF EarthCube end user workshop was held at the USC Wrigley Marine Science Center on Catalina Island, California in August 2013. The meeting brought together a group of scientists with experience in ocean science, environmental genomics and allied sciences, biological oceanography, bioinformatics and computer science, as well as NSF and private Foundation program managers. A main goal of the Ocean Omics NSF EarthCube end user workshop was to help identify and prioritize a set of scientific drivers and cyberinfrastructure requirements necessary to enable the storage, curation, federation, and comparative analyses of large and small scale ocean science genomic, metagenomic, metatranscriptomic and metaproteomic datasets that are rapidly accumulating. Although the collection, availability and analyses of these and similar datasets are improving our understanding of ecosystem processes and predicting their future trajectories, the necessary computational and analytical tools and infrastructures to manage, share, analyze and visualize them needs accelerated development and expansion. Workshop participants discussed these current challenges, and identified specific tools, technologies and infrastructures that will be required to continue advancing ‘omics applications in ocean science biology, marine biogeochemistry marine biology, and biological oceanography in the 21^st^ century.

## Background and purpose of the meeting

The NSF EarthCube initiative was launched in June 2011 to seek “transformative concepts and approaches to create integrated data management infrastructures across the Geosciences.” NSF and a community of U.S. geoscientists and cyberscientists have recognized that “for EarthCube to achieve its potential as a new data and knowledge management system for the 21st Century, the collective needs and desires of geoscientists across the disciplines must be made known so similarities and difference between user groups and disciplines can be identified and addressed.” To this end, the NSF Geosciences Directorate solicited proposals to conduct domain workshops “designed to listen to the needs of the end-user groups that make up the geosciences and associated research groups and to understand better how data-enabled science can help them achieve their scientific goals.”

The overall purpose of the August 2013 Catalina end user workshop was to develop and articulate a set of unifying scientific and computational requirements shared by ocean ‘omic scientists. Participants were challenged to envision new ways to integrate the community’s data collection, archiving and analyses, and scientific efforts, from the perspectives of both domain-specific ocean scientists as well as computer scientists. The workshop participants discussed the available and existing suite of tools and technologies available to perform the large scale ‘omics experiments and analytics, identified gaps in existing infrastructures, and attempted to forecast potential future directions for these fields.

**The specific goals of the Ocean ‘Omics Workshop were to:**
1. Identify the critical scientific challenges and cyberinfrastructure constraints for ocean ‘omic science.
2. Develop a set of relevant ocean ‘omic science use-cases that identity and combine compelling science drivers with explicit cyberinfrastructure needs.
3. Identify the data management, analytical and associated cyber-infrastructure capabilities required to address the critical ocean ‘omic scientific challenges, both current and future.

### Participants

The participants (see Participant List) were invited based on: 1) their scientific and technical experience and interest in the scientific questions challenges in the context of ocean ‘omics science; and 2) their knowledge of cyberinfrastructure technologies, applications, and current capabilities, in the context of ocean ‘omics science and ‘omics in general.

## Addressing meeting goals: Outputs and Conclusions

### I. Critical scientific challenges and cyberinfrastructure constraints

There are many challenges that a community must face if it is to design and implement high impact interdisciplinary science. Primary among these is communication, with the need to develop a common language to minimize misunderstanding and misinterpretation when discussing project design, implementation and analyses. Currently, there exist a number of different databases for exploring metagenomic, other ‘omic, and environmental datasets in the context of ocean science ([4-7]). However, a common language to facilitate communication must be built on a series of standardization efforts. The internet is a prime example of this, whereby all computers used standard languages to facilitate exquisitely integrated interactions across the world, enabling communication between myriad disciplines. However, it is still a challenge for any community to develop, validate and implement standardized and federated procedures for sample collection schemes, sample QC/QA, data formats, annotation workflows, and data analyses. Even more complex is the task of integrating those with geochemical, biological and physical oceanographic data over multiple nested spatiotemporal scales, to allow researchers from different scientific disciplines to interact and actually use the data being generated. Grassroots efforts such as the Genomic Standards Consortium [8]; have overseen the development of standard formats and languages for describing how sequencing data was generated and for capturing the contextual environmental data (physical, chemical and biological data streams) in a common, machine-readable format. These efforts are perceived widely as facilitating data sharing, and data re-use, by limiting the need for detailed literature searches, and enabling meta-analyses of existing data resources (in this case genomics) for generating novel high-impact science. However, these efforts are still limited in their scope and despite considerable work and integration with public databases for sequence data (e.g. INSDC, MGRAST, IMG/M, CAMERA, etc.), uptake and incorporation by the community takes time, and is currently still limited. There are a number of reasons for the slow adoption of community-wide standards and practices, briefly explored below.

A primary concern, raised in the workshop was the lack of access that the community has to data storage space, and transfer mechanisms for the sharing and archiving of raw data, processed data, data products from workflows, and records of the provenance of data analyses. This concern is compounded by the limited access to large scale, high performance compute capabilities necessary for the annotation, comparison, statistical analyses and other workflows required for analyses of large scale ocean 'omic datasets. Even with common languages to describe and share sequence data that could aid interaction in the absence of any technical impediment, there is a continued need for the development of these standards as new sequence types, and non-sequence-based data types (e.g. mass spectrometry used in proteomics and metabolomics) emerge, that also will need to be stored, accessed and analyzed and federated with other environmental and 'omic data streams.

Currently, the community also lacks sufficient tools for analysis and simultaneous visualization and inter-comparison of heterogeneous data types (e.g., environmental, 'omic and oceanographic datasets). This concern is also a primary factor limiting the integration of emerging 'omics datasets and analyses with existing and developing physical and biogeochemical models. This is partly an analytical problem (e.g., the mapping of genes and pathways onto their respective biogeochemical activities), and partly an integration problem, requiring the combination of quantitative 'omics-derived biogeochemical information, with quantitative geophysical and geochemical models. The development of better analytical and visualization tools, and modeling platforms to capture translation knowledge must come from the community, and be driven by community need so as to ensure that these products are both relevant and up-to-date. However, focus and funding for developing these tools must still come from the agencies, since the‘cool tools’ that we take for granted (iphone apps, facebook, professional software platforms, etc.) will always have a shelf life, and lack the interface which enables researchers to overcome technical education barriers to use. Facilitating the development of both the software tools that improve analysis and visualization of ocean omic datasets and of the platforms that facilitate integrated modeling of diverse data streams is essential if we are to fully capitalize on existing investment in current research. However, this will also take both innovation and sustained investment, along with a certain degree of community consensus on the existing tool infrastructure that is required to ‘do the job right’. A related issue is the efficient distribution and dissemination of bioinformatics tools. Often these tools are developed in individual laboratories without intuitive user interfaces and in formats or with dependencies on other software that hinder their utilization by the broader community. Development of procedures, best practices, and infrastructure to facilitate the dissemination of such tools is required to capture and coordinate community-driven advances in analytical capabilities.

The majority of our community is dispersed through academic and federal labs that differ vastly with regards to institutional resources for empowering large scale computing. Major advances for elucidating meaningful interpretations of ‘omics data will require investments in computing and informatics infrastructure that can be utilized and adapted by users regardless of institutional access. If resources don’t become available across the community, we will have institutional winners and losers, whereby the scientific home of a researcher or student will largely dictate their ability to work with ‘omic scale data.

## II. Ocean ‘omics science grand challenges and questions: current and future

The rapidly increasing throughput and declining costs of producing ‘omics data offers new opportunities to address pressing issues in ocean sciences. Several high-priority science questions were identified that hold promise for significant advances through application of omic approaches and that will likely be the focus of interdisciplinary efforts during the next 5-15 years. Several science questions and challenges were identified as promising use case scenarios, that combine compelling science drivers with explicit cyberinfrastructure needs.

### Science Question and Challenge # 1

*“How do biological population structure and function co-vary with physical and chemical oceanographic parameters within and between different oceanographic provinces?”* The physical and chemical environment shapes the structure and function of marine microbial communities, and microbial communities in turn influence the chemistry of the seas. Over the past five years, it has become possible to deeply characterize diverse microbial communities at the genomic level and to track the expression of numerous genomes across space and time. At least from a data acquisition standpoint, we are now poised to address questions such as:

How do steep physical and chemical gradients result in steep microbial functional gradients and drive changes in microbial biodiversity?
How do microbial communities in the ocean fluctuate across key boundaries and gradients, such as distance from land, seafloor spreading centers, gyres, and upwelling zones?
How do microbial communities change as a function of geochemistry, currents, and crustal age?
How do microbial community dynamics affect the flux of matter and energy throughout the ocean’s water column, benthos and subsurface?
One of the greater challenges in addressing the above questions is to rapidly generate, analyze, annotate and make publically accessible the rapidly accumulating new, large scale omic datasets and metadata. Another choke point is the availability of genomic data for key organisms, that is generally limited to what has been published in GenBank. As such, researchers wishing to map their transcriptomic data against available genomes will be limited to what is available at any given time. Furthermore, the cycle time and compute resources available for analyses are also limited. Publishing of further resources in the public domain, and placing these data resources in cloud computing infrastructure (for both storage and analytical purposes), will greatly facilitate answering these questions.

### Science Question and Challenge # 2

“*What are the underlying molecular and biochemical mechanisms that regulate the physiological responses of microbes to environmental change, and their downstream biogeochemical consequences and feedbacks?”* The capacity to deeply track the content and expression of microbial genomes across space and time provides windows into the genetic responses of microbes to environmental change. Such dynamics can be observed both in the laboratory and in the field. In the next 5-10 years, as ocean ‘omics datasets continue to grow in temporal and spatial coverage, there will be increasing and emergent opportunities for meta-analyses that characterize responses of microbes to environmental perturbation. One can now envision ‘omics data resolving longer-term microbial responses, such as dynamics on decadal time scales, in much the same way that large-scale physical and chemical data currently provide pictures of climate change. In some cases these insights may uncover well-known organisms, pathways, or genes, while in other cases an observational approach may highlight unknown players (organisms, pathways, or genes) as key responders to perturbation and mediators of feedbacks. Hence, if the data is effectively preserved and archived, ‘omic datasets could represent powerful means of discovery and hypothesis generation. Central science questions here include:

What are the underlying molecular and biochemical mechanisms that regulate the physiological responses of microbes to environmental change, and their downstream biogeochemical consequences and feedbacks?
How does 'omic and population plasticity in microbes bolster ecosystem resilience to disturbances?
How do global change and environmental disturbance impact genomic repertoires, transcriptional organization, protein and metabolome content, and biogeochemical activity?
Which microbial taxa and processes are affected by rapid polar climate change, and how do those taxa impact the budget of greenhouse gases, permafrost thawing and dissolved organic carbon release and transport in time and space?

### Science Question and Challenge # 3

*“Can 'omics data be used to describe and model ecosystem processes and their trajectories?“* To date, omics information has largely been utilized to uncover specific populations that underpin key processes, hence deepening our understanding of microbial communities and the ecosystem processes they mediate. A major opportunity (and challenge) for the future is to better interpret this information so that it can be leveraged to predict future trajectories of large, microbially-mediated ecosystem processes. For example, accurate mapping of microbial genes and gene products onto the cognate biogeochemical cycles they catalyze, could enable further modeling based on gene distributions. Such gene to biogeochemical reaction associations have potential to link microorganisms to their activities in specific environmental settings. Such distributions can be used to generate hypotheses about the nature of biogeochemical feedback loops, and their possible variability under different scenarios of climate and biogeochemical change. Omics data is valuable for both the parameterization of models (e.g., defining the range of different microbial functional groups and traits that would be useful to simulate), as well as for the validation and tuning of models by comparing model outputs to ‘omics observations and biogeochemical process measurements.

Although there are still many barriers to surmount, it is now possible to imagine the development of integrated ‘omic-biogeochemical-ecological models that could be utilized by stakeholders and regulators for the effective management and monitoring of water and ecosystem resources such as fisheries. One of the most obstructive barriers is access to multiple data types (environmental data, time series data, organismal distributions and their variability, process measurements, omics datasets, etc.) that are needed to drive predictions. Researchers require access to ‘omics data, but also biogeochemical, physical, remote sensing data as well. These data types are often generated by specialists and the formats are not interchangeable, driving the need to for more cross talk among different disciplines. Underlying science questions here include:

How can 'omics data be more effectively leveraged into predictive frameworks for understanding ecosystem processes and their future trajectories?
How can 'omics data be better interpreted and analyzed using graphical outputs, models and indicators, that would be useful to managers and stakeholders for efficiently monitoring ecosystem changes and their consequences?

## III. Data management, analytical and associated and cyber-infrastructure capabilities required to meet critical scientific challenges, current and future.

The attendees of the workshop represented a broad representation of the community of users and developers; as such these tool recommendations stem largely from individual experience across a continuum of disciplinary expertise.

In the context of the science questions and use cases discussed above, a number of requirements and needs for cyberinfrastructure can be identified. Five categories were identified as being of immediate importance to improve the archiving of and access to data resources, their analyses, exploration, and visualization, and their integration between microbial genomics, zoology, oceanography, biogeochemistry and other overlapping disciplines:

1. *Development of integrated omics databases* is required to enable curation, maintenance and data standardization, to facilitate primary data submission, extraction of raw and processed data, and intelligent query of data-resources. Achieving this will require tools for rapid and simple data query and metadata association. While these do exist, they are not suitable for the community’s needs. In part, this is because they were developed without community-wide consultation during development. Building community concensus is an arduous and complicated process, with its own downsides. Integration and tool development should incorporate non-sequence-based datasets (e.g. metabolomics and lipidomics) into existing/emerging oceanographic 'omics database/analysis/visualization platforms. Environmental 'omic databases need to be:
  (a) federated (i.e., all datasets can be interoperably queried and transparently accessible)
  (b) curated (validated and updated, as for example NCBI RefSeq datasets)
  (c) sustained (i.e. a five-year commitment of support will not provide sustainable infrastructure), and importantly
  (d) intuitively accessible to a broad range of scientists, and the public.
2. The ocean 'omics community would benefit from “Google-like” or “Kayak-like” search and suggestion functions and engines, that could query across complex and heterogeneous, federated environmental, oceanographic and 'omic databases. However, as highlighted above this will require significant and sustained investment and development.
3. Tools and procedures are required for access to high performance computing and statistical analyses of large scale 'omic datasets, that could accommodate both naïve users as well as experienced “power users”. One possibility is a user facility that functions similarly to the UNOLS oceanographic facilities, that would provide access to software developers, bioinformaticians, and analytical tools, as well as the hardware (storage facilities, servers, clouds, etc) required for 'omic analyses. Researchers could request access to this facility in association with successful grant applications, as with UNOLS. Extending the capabilities of BCO-DMO or similar services is an alterative approach. This framework could also be an efficient means of connecting biologists and oceanographers to bioinformaticians for the purpose of tool development, perhaps through a special streamlined application process such as those used at national laboratories (e.g., synchrotron sources).
4. Tools are required for more intuitive, accessible and integrated visualization of linked environmental, 'omic and oceanographic (and other interdisciplinary) data sets. Statistical tools and techniques for dataset inter-comparison and spatiotemporal modeling also are critical and need considerable development to manage the scope and scale of both existing and future datasets.
5. The community would benefit from access to a web clearinghouse/portal with links to standard “ocean 'omics” best practices, algorithms, software, tutorials, forums, and workflows, as well as analytical and statistical methods under development, with entry points for both naïve and power users, would be a useful resource for the community. Such a resource could also facilitate and incentivize the effective dissemination, maintenance, and improvement of bioinformatic tools.

## Ocean ‘omics meeting recommendations: next steps

The workshop attendees discussed some of the necessary first steps and enabling activities that will help move ‘ocean omics science, technology and education into the future.

1. Cross train and educate computer scientists and engineers, and ocean and earth scientists to improve communication and collaboration among disciplines. This includes training and education to develop cross-disciplinary expertise within and between bioinformatics, the Earth sciences, and the Ocean sciences.
2. Facilitate access, availability and utilization of NSF supercomputers for the Earth and Ocean sciences communities. Using government supercomputers should be as technically easy, and as feasible as accessing the Amazon EC2 grid, especially in regard to requesting and accessing compute cycles.
3. Plan and initiate a community Research Coordination Network to support cyberinfrastructure technology and infrastructure development and education in ocean 'omics.
4. Promote the development of an EarthCube system that would combine the facilitative role of the BCO-DMO database (or similar), with novel and flexible analytic and visualization services for exploring ocean ‘omics oceanographic data (e.g., Ocean Data View-like software and tools, for ocean ‘omics data).
5. Further identify ocean 'omics cyberinfrastructure “parts” (e.g. dataset curators, search engines, high performance compute facilities, workflows, user analytical facilities, developers, etc.) that are operational and in use now, and determine which ones might be further improved, developed, federated, and networked into a functional EarthCube community ocean 'omics cyberinfrastructure solution.

**Table:** Participant list

First name	Surname	Institution
Andy	Allen	J. Craig Venter Institute
Eric	Allen	University of California San Diego
Jean-Paul	Baquiran	University of Southern California
Doug	Bartlett	University of California San Diego
Alison	Buchan	University of Tennessee
Lisa	Campbell	Texas A&M University
Doug	Capone	University of Southern California
Marian	Carlson	Simons Foundation
David	Caron	University of Southern California
Cyndy	Chandler	Woods Hole Oceanographic Institution
Dylan	Chivian	Lawrence Berkeley National Laboratory
Byron	Crump	Oregon State University
Ed	DeLong	M.I.T. & University of Hawaii, Manoa
Sonya	Dhyrman	Columbia University
Greg	Dick	University of Michigan
Katrina	Edwards	University of Southern California
Mark	Ellisman	University of California San Diego
Emiley	Eloe	Gordon and Betty Moore Foundation
Jed	Fuhrman	University of Southern California
Dave	Garrison	U. S. National Science Foundation
Jack	Gilbert	Argonne National Laboratory
John	Heidelberg	University of Southern California
Julie	Huber	Marine Biological Laboratory
Matt	Janicak	University of Southern California
Bethany	Jenkins	University of Rhode Island
Nikos	Kyrpides	Lawrence Berkeley National Laboratory
Michael	Lee	University of Southern California
Abel	Lin	University of California San Diego
Karen	Lloyd	University of Tennessee
Katherine	Mackey	Woods Hole Oceanographic Institution
Vanessa	Michelou	University of Hawaii, Manoa
Bob	Morris	University of Washington
Jasmine	Nahorniak	Oregon State University
Craig	Nelson	University of Hawaii, Manoa
Anton	Post	U.S. National Science Foundation
Gustavo	Ramirez	University of Southern California
Barbara	Ransom	U. S. National Science Foundation
Mike	Rappe	University of Hawaii, Manoa
Frank	Stewart	Georgia Institute of Technology
Gowtham	Subbarao	University of California San Diego
Andreas	Teske	University of North Carolina
Kim	Thamatrakoln	Rutgers University
Ben	van Mooy	Woods Hole Oceaographic Institution
K. Eric	Wommack	University of Delaware
Lisa	Zeigler Allen	J. Craig Venter Institute
Erik	Zinser	University of Tennessee
